# Supplementary material for: Immunomodulatory effects of nanoparticles on dendritic cells in a model of allergic contact dermatitis: importance of PD-L2 expression
Source: Sci Rep. 2023 Sep 25;13:15992. doi: 10.1038/s41598-023-42797-5 (PMC10520013; doi:10.1038/s41598-023-42797-5)
Supplement: Supplementary file 1 — Supplementary Information. [file 41598_2023_42797_MOESM1_ESM.docx]

Supplementary Materials

**Immunomodulatory Effects of Nanoparticles on Dendritic Cells in a Model of Allergic Contact Dermatitis - importance PD-L2 expression**

Angela Wong Lau et al.


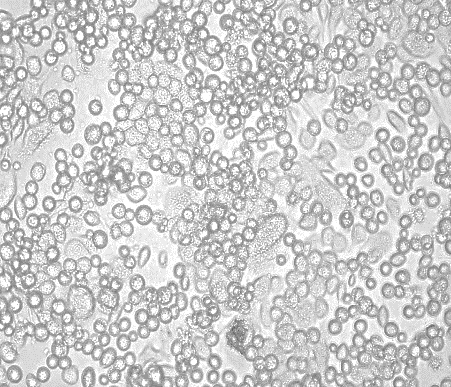

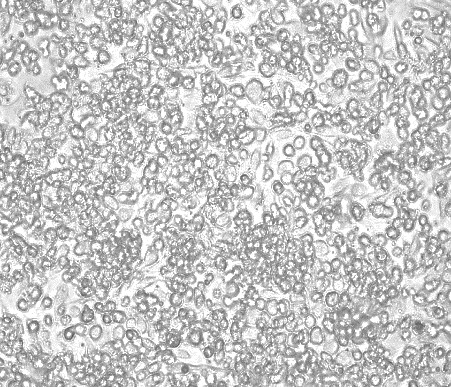

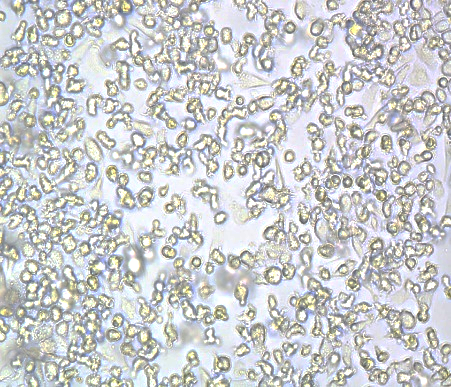

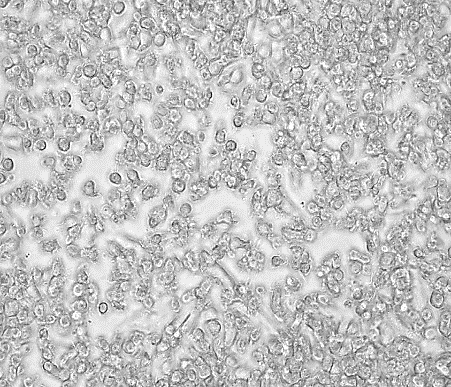

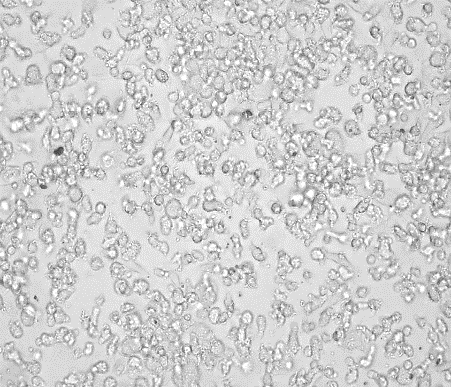


**50µm**

**A**

**B**

**C**

**D**

**E**

**50µm**

**50µm**

**50µm**

**50µm**

**Fig. S1.** **Bright field images of each treatment after 1 hr exposure, taken in 20x.**(**a**) untreated immature BMDCs show a heterogeneous populations (adherent and semi-adherent) (**b**) BMDCs exposed to 0.001 mM DNFB show some semi-adherent cell population becoming round (**c**) BMDCs exposed to 0.01 mM DNFB show both adherent and semi-adherent cell populations becoming significantly round (**d**) BMDCs exposed to 0.01 mg/mL SiO_2_ NPs show adherent and semi-adherent cell populations keeping its cell morphology (**e**) BMDCs exposed to 0.005 mg/mL mTiO_2_ NPs show adherent and semi-adherent cell populations keeping the dendritic cell morphology.


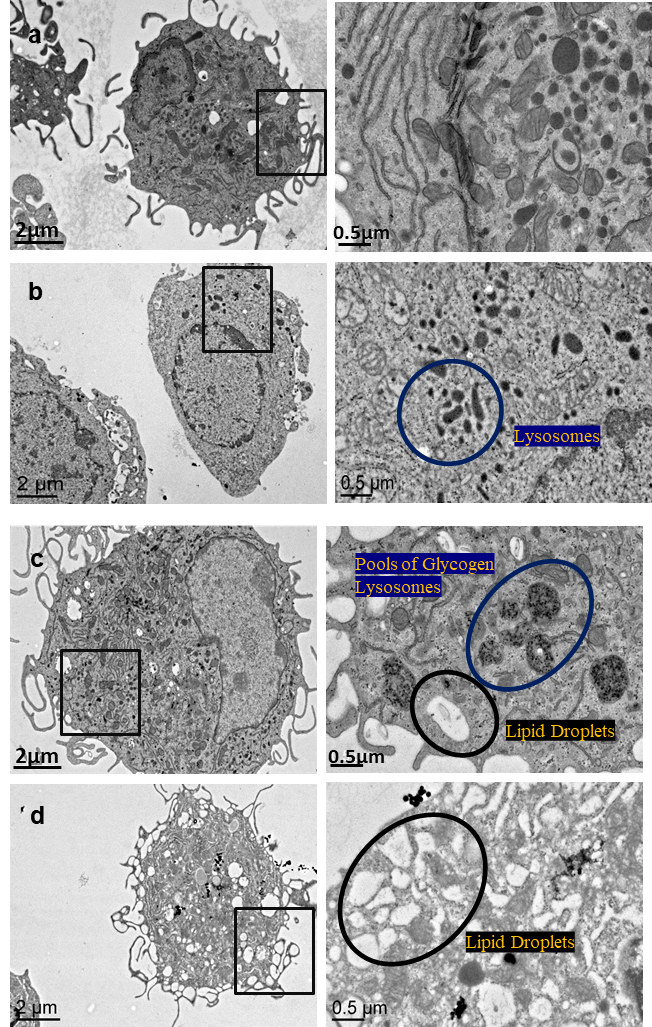


**Fig. S2. TEM images of BMDC after 1 hr exposure of each treatment in 2 μm and 0.5 μm scale.**(**A**) untreated immature BMDCs illustrating the characteristic dendrites (**B**) BMDCs treated with 0.01 mM DNFB show loss of dendrites (**C**) BMDCs treated with 0.01 mg/mL SiO_2_ NPs retain their dendrites and there is an increased glycogen granules (**D**) BMDCs treated with 0.005 mg/mL mTiO_2_ NPs retain their dendrites and there is an increase in lipid droplets. It is unknown at this time if the rounding of cells (loss of dendricity) is a toxicity response or an activation response of the cells to retract dendrites to migrate out of peripheral tissues to lymph nodes.


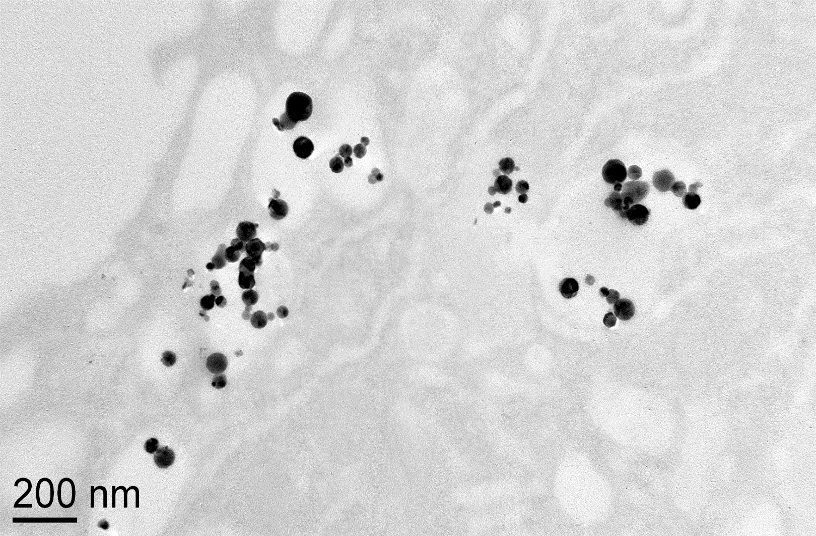

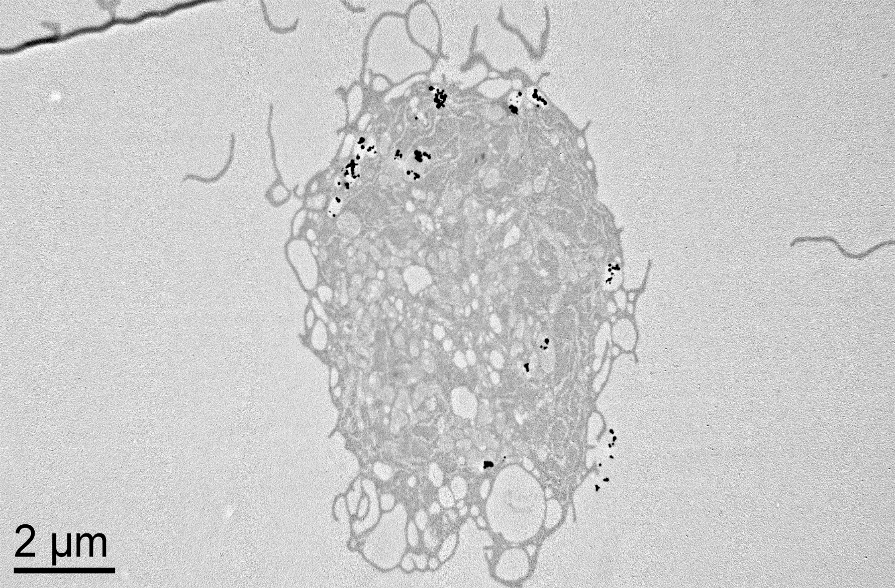





Fig. S3. Characterization of mTiO_2_ NPs by TEM. BMDCs were culture with mTiO_2_ NPs (0.005 mg/ml) for 1 hr. Cells were then prepared for TEM to assess NP uptake. Results show endocytosed mTiO_2_ NPs with an average size of 51.6 +/- 12.5 nm. Taking the ratio of the standard deviation to mean particle size the polydispersity is 0.24, which is similar to the PDI of 0.296 that was measured by DLS with the particles suspended in water that had a hydrodynamic ratio of 556.4 nm +/- 33.6 nm^1^. Also shown is a TEM image of mTiO_2_ suspended in water and dried onto a TEM grid. Results show a tendency to form clusters whereas, they are more dispersed and rounded inside the BMDCs. Mn is highly redox reactive and soluble, hence the biotransformation of mTiO_2_ in cells is expected.


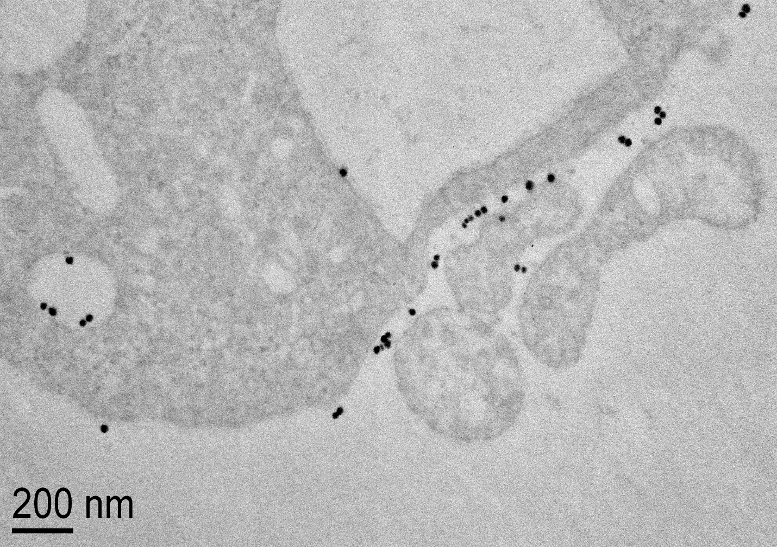

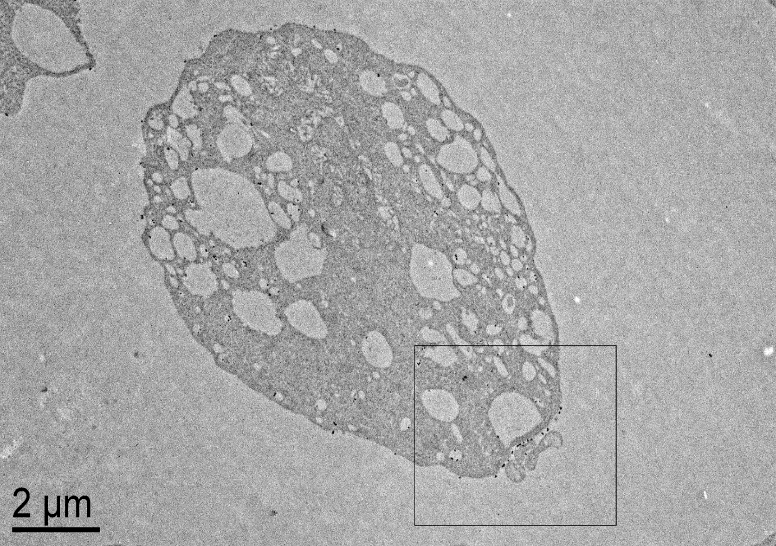


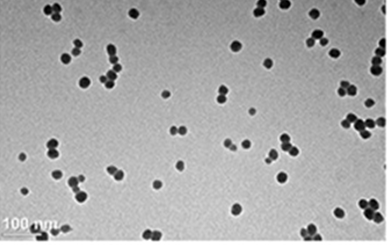


Fig. S4. Characterization of SiO_2_ NPs by TEM. BMDCs were culture with DNFB (0.01 mM) plus SiO_2_ (0.01 mg/ml) for 1 hr. Cells were then prepared for TEM to assess NP uptake. Results show endocytosed SiO_2_ NPs with an average size of 20.6 +/- 3.5 nm. Taking the ratio of the standard deviation to mean particle size, the polydispersity is 0.17, which is slightly lower than the PDI of 0.236 that measured by DLS with the particles suspended in water that had a hydrodynamic ratio of 33.5 nm +/- 3.3 nm^2^. Also shown is a TEM image of SiO_2_ NPs suspended in water and dried directly on the TEM grid.


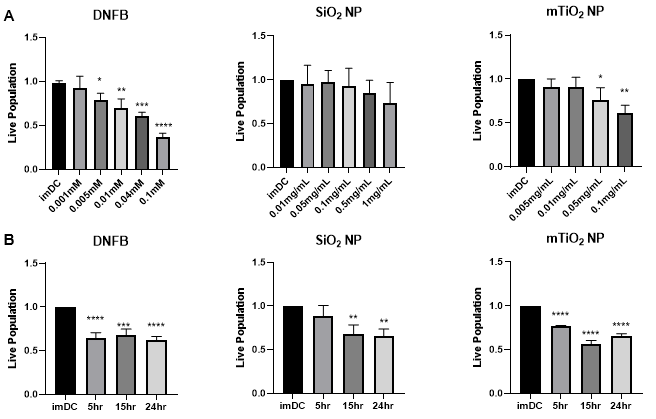


**Fig. S5.** **Effects of DNFB, SiO_2_ and mTiO_2_ exposure on BMDC cytotoxicity as a function of concentration and time measured with PrestoBlue assay.**BMDCs were harvested on day 8 and treated with DNFB, SiO_2_ and mTiO_2_ to study cytotoxicity as a function of concentration and time using PrestoBlue. (**A**) Cell viability as a function of concentration for a 1 hr exposure. (**B**) Cell viability following exposure to the lowest non-cytotoxic concentrations from (**A**) and exposed over a period of 5 hr, 15 hr and 24 hr. Results indicate that cytotoxicity of DNFB and mTiO_2_ NPs on BMDCs was dose- and time- dependent. Live population was normalized the imDC no treatment control. Ordinary one-way ANOVA was performed and compared to imDC. N=3-5. Mean ± SD. *p < 0.05, **p < 0.01, ***p < 0.001, ****p < 0.0001.

**B**

**A**

**C**

**Fig. S6. Supernatants were collected to analyze cytokine production by ELISA for IL-6, TNFα and IL-10 as a function of concentration for 1 hr exposure.**BMDCs were exposed to increasing concentrations of each stressor for 1 hr. (**A**) DNFB which upregulated IL-10 only at the highest concentration (**B**) SiO_2_ NPs upregulated IL-6 (**C**) mTiO_2_ NPs upregulated IL-6, TNFα and IL-10. Concentration was normalized against % of live cells. Ordinary one-way ANOVA was performed and compared to untreated imDC. N=3-5. Mean ± SD. *p < 0.05, **p < 0.01, ***p < 0.001, ****p < 0.0001.

**Fig. S7. Supernatants were collected in order to analyze secreted cytokine production following LPS exposure over time.**BMDCs were exposed to LPS (50 ng/mL) on day 8 and the supernatants were collected to analyze production of IL-6, TNFα and IL-10 by ELISA over time. LPS is a strong, potent stressor that upregulated IL-6, TNFα and IL-10 over time. Ordinary one-way ANOVA was performed and compared to imDC. N=3-5. Mean ± SD. *p < 0.05, **p < 0.01, ***p < 0.001, ****p < 0.0001.

**Fig. S8. Intracellular flow cytometry staining of BMDCs at increasing concentrations for 1 hr exposure.**BMDCs were treated with increasing concentrations of DNFB, SiO_2_ and mTiO_2_ for 1 hr and intracellularly stained for IL-10 and TNFα. SiO_2_ NPs upregulated IL-10 at high concentrations without upregulating TNFα while the opposite occurs with mTiO_2_ NPs. Ordinary one-way ANOVA was performed and compared to imDC. N=3-5. Mean ± SD. *p < 0.05, **p < 0.01, ***p < 0.001, ****p < 0.0001.

**Fig. S9. Intracellular flow cytometry staining of BMDCs over time.**BMDCs were treated to the lowest concentrations of each stressors (0.001 mM DNFB**,** 0.01 mg/mL Si 20 nm, and 0.005 mg/mL mTiO_2_) and intracellularly stained for IL-10 and TNFα. mTiO_2_ upregulated TNFa and IL-10 while SiO_2_ NPs only upregulated IL-10. Ordinary one-way ANOVA was performed and compared to untreated imDC. N=3-5. Mean ± SD. *p < 0.05.


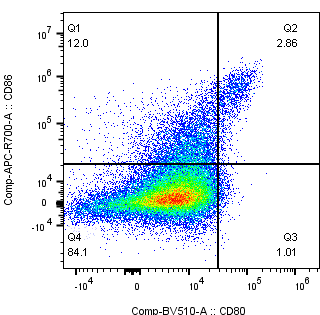


**CD86 vs CD80**


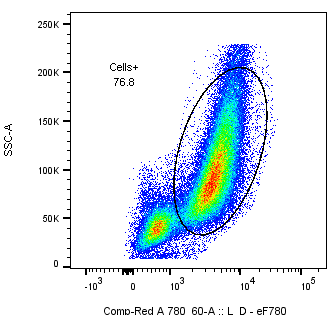


**Cells +**

**Debris**


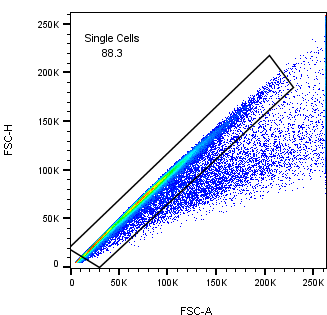

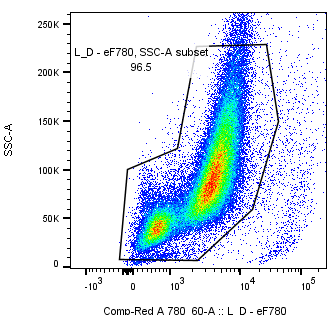

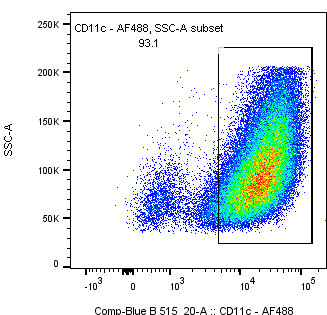

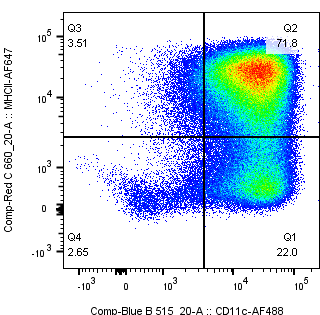


**Single Gating**

**MHCII vs CD11c**

**Double Gating**

**CD11c**

**CD11c+MHCII+**

**Single Cells**

**Dead Cells Exclusion**

**Live Cells**

**A**

**B**

**C**

**D**

**E**

**F**

**Fig. S10A.** **Gating strategy used to differentiate BMDC cell populations performed by FlowJo.**(**A**) Single cells were gated. (**B**) Fixable Viability Dye eFluor™ 780 was used to separated live cell population from the dead cell population. (**C**) The cell population excluding the debris (lower left corner) was selected. (**E**) Cells were gated under CD11c+MHCII+ (Q2.) which we defined as the BMDC. (**F**) the BMDC were gated under CD80/CD86 subpopulations split into four quadrants (Q1., Q2., Q3. and Q4.) giving us information about the CD86 and C80 single positive cells, the naive double negative (DN) and activated double positive (DP) subpopulations.


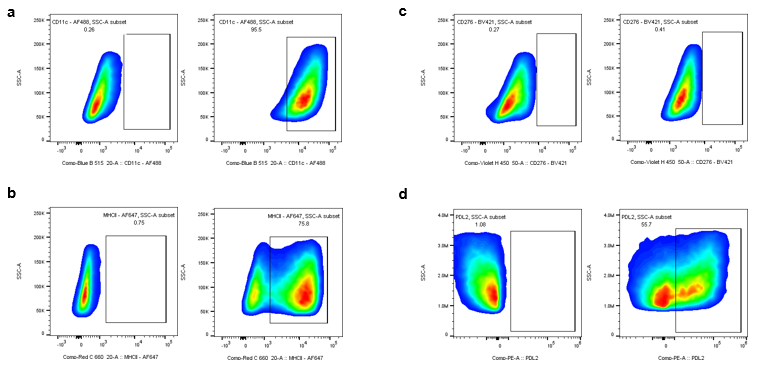


**Fig. S10B. Example of FMO controls for untreated imDCs.**

BMDCs were cultured in complete media (RPMI) for 8 days. Flow cytometry was done to test for the expression of activation markers. The markers selected for example shown with the FMO controls are: (a) CD11c, (b) MHCII, (c) CD276 and (d) PD-L2


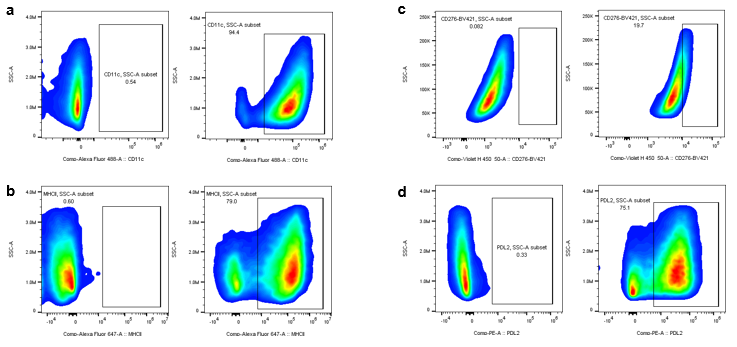


**Fig. S10C. Example of FMO controls for BMDC treated with 0.005 mM DNDB for 1 hr.** BMDCs were cultured in complete media (RPMI) for 8 days. Then they were cultured for 1 hr with 0.005mM DNFB. Flow cytometry was done to test for the expression of activation markers. The markers selected for example shown with the FMO controls are: (a) CD11c, (b) MHCII, (c) CD276 and (d) PD-L2

**Fig. S11. Changes in B7 ligands following exposure of BMDCs to LPS over time for single gating on live cells.**
Phenotypic characteristics of BMDCs following LPS (50 ng/mL) exposure over time using single gating (entire live cell population) of B7 co-stimulatory molecules. Exposure of LPS downregulated the expression of CD11c and upregulated the expression of MHCII, CD86, CD80, PD-L1 and no changes in PD-L2 were evident. CD276 increases early (3-6 hr) and decreases >15 hr. Ordinary one-way ANOVA was performed and compared to untreated imDC. N=3-5. Mean ± SD. *p < 0.05, **p < 0.01, ***p < 0.001, ****p < 0.0001.

**Fig. S12. Changes in B7 ligand expression on CD11c+MHCII+ BMDCs (double gating) following exposure to LPS over time.** Phenotypic characteristics of BMDCs following LPS (50 ng/mL) exposure over time using double gating (CD11c+MHCII+) of B7 co-stimulatory molecules. Exposure of LPS upregulated the expression of CD80, CD86 and PD-L1 with time and no changes in PD-L2 were evident. CD276 increases early (3-6 hr) and decreases >15 hr. These trends are consistent with gating on all cells **Fig. S10**. Ordinary one-way ANOVA was performed and compared to imDC. N=3-5. Mean ± SD. *p < 0.05, **p < 0.01, ***p < 0.001, ****p < 0.0001.

**Fig. S13 Co-stimulatory molecules from the B7 family quantified by flow cytometry under CD80/CD86 single positive subpopulations.**

Results for DNFB are colored in blue, SiO_2_ in green and mTiO_2_ in purple. CD80/CD86 subpopulations were divided and CD276 (B7-H3), PD-L1 (B7-H1) and PD-L2 (B7-DC) were gated under singles positives CD86-CD80+ (Q1.) or CD8+CD80-(Q3.) subpopulations, in order to determine whether their expression varies under the different activation states of BMDCs by using the activation markers CD80/CD86 following single exposures. The lowest non-cytotoxic concentrations of each stressor 0.001 mM DNFB, 0.01 mg/mL SiO_2_ NPs and 0.005 mg/mL mTiO_2_ NPs were used to treat the cells over 5 hr, 15 hr, and 24 hr. There was a decrease due to upregulation of CD80+ single positive cells on CD80/CD86 subpopulations. There were similarities between DNFB and mTiO_2_ on PD-L1 expression where single positives subpopulation increases. On the other hand, SiO_2_ NPs showed a downregulation of PD-L1 expression. PD-L2 showed a decrease in the CD86 single positive cells. Expression of CD276 was only upregulated on CD80+ quadrants (Q3.). A two-way ANOVA was performed and compared to imDC. N=3-5. Mean ± SD. *p < 0.05, **p < 0.01, ***p < 0.001, ****p < 0.0001.

**A**

**B**

**C**

**D**

**E**

**F**

**G**

**H**

**Fig. S14. Cytotoxicity and cytokines for co-exposure of DNFB with SiO_2_ or mTiO_2_ NPs for 1 hr.**
BMDCs were subjected to the co-exposure of the DNFB hapten with the SiO_2_ or mTiO_2_ NPs after 1 hr. Cell viability, based on flow cytometry, of BMDCs was compared. (**A**) SiO_2_ NPs exhibited cytoprotective effects while (**B**) mTiO_2_ NPs had no effect. Supernatants were then collected in order to analyze cytokine production by ELISA assay for (**C-D**) IL-6 which showed that addition of mTiO_2_ increased its secretion. (**E-F**) addition of either NPs increased the production of TNFα and (**G-H**) both NPs had no effect on IL-10 secretion. Ordinary one-way ANOVA was performed and compared to imDC (*) and DNFB (#). Concentration was normalized against % of live cells. N=3-5. Mean ± SD. */#p < 0.05, **/##p < 0.01, ***/###p < 0.001, ****/####p < 0.0001.

**Fig. S15 Co-stimulatory molecules from the B7 family quantified by flow cytometry under CD80/CD86 subpopulations following 1 hr co-exposure.**
CD80/CD86 subpopulations were divided and CD276 (B7-H3), PD-L1 (B7-H1) and PD-L2 (B7-DC) were gated under double positive (DP) population (Q2.) or double negative (DN) population (Q4.) to determine whether their expression varies under the different activation states of BMDCs using the CD80/CD86 markers following exposure to DNFB alone (0.01 or 0.001 mM) or co-exposure with 0.01 mg/mL SiO_2_ or 0.005 mg/mL mTiO_2_. Most sticking effects of the NPs occur in the DNFB induced changes in PD-L1 and PD-L2. SiO_2_ decrease PD-L1 where as TiO_2_ co-exposure increases it in the CD80+CD86+ population. TiO_2_ co-exposure decreases PD-L2 expression in the CD80+CD86+ whereas SiO_2_ has no effect. A two-way ANOVA was performed and compared to imDC (*) and DNFB (#). N=3-5. Mean ± SD. **/##p < 0.01, ***/###p < 0.001, ****/####p < 0.0001.

Fig. S16. BMDCs profile on Day 8.
Graphic representation of murine BMDCs profile on day 8 with the markers F4/80 (macrophages), CD11b and CD11c (DCs) and MHCII. The graphs show mean ± SD. F4/80 (n=5), CD11b (n=8), CD11c (n=10), MHCI (n=10), CD3 (n=1), CD19 (n=1).

**Table S1.** Flow panel with its corresponding fluorochrome, concentration, clone number, supplier, and catalog number

| **Antibody** | **Concentrations (µg/mL)** | **Fluorochrome** | **Clone #** | **Supplier** | **Cat #** |
| --- | --- | --- | --- | --- | --- |
| CD11c | 0.5 | Alexa Fluor 488 | N418 | BioLegend | 117311-BL |
| CD11b | 0.25 | PE-eFluor™ 610 | M1/70 | Invitrogen | 61-0112-82 |
| MHCII | 0.3 | Alexa Fluor 647 | M5/114.15.2 | BioLegend | 107618-BL |
| CD86 (B7-2) | 0.5 | APC-R700 | GL1 | BD Biosciences | BDB565479 |
| CD80 (B7-1) | 0.15 | Brilliant Violet 510 | 16-10A1 | BioLegend | BDB740130 |
| CD276 (B7-H3) | 0.08 | Brilliant Violet 421 | MIH32 | BD Biosciences | BDB562634 |
| PD-L1 (B7-H1, CD274) | 0.4 | Brilliant Violet 711 | 10F.9G2 | BioLegend | 10759-272 |
| PD-L2 (B7-DC, CD273) | 0.2 | PE | TY25 | Invitrogen | 12-5986-82 |
| TNFα | 0.1 | PE-Cyanine7 | MP6-XT22 | Invitrogen | 25-7321-82 |
| IL-10 | 0.2 | PerCP-Cyanine5.5 | JES5-16E3 | Invitrogen | 45-7101-82 |
| F4/80 | 0.2 | PE-Cy5 | BM8 | BioLegend | 123112-BL |
| CD19 | 0.3 | APC Cy7 | SJ25C1 | BD Biosciences | 557791 |
| CD3 | 0.3 | AF700 | SP34-2 | BD Biosciences | 557917 |
| FVD | 0.6 | eFluor 780 |  | Invitrogen | 65-0865-14 |
| FcBlock | 0.5 | Antigen: CD16/32 | 93 | BioLegend | 101320-BL |

1 Palmer, B. C. & DeLouise, L. A. Morphology-dependent titanium dioxide nanoparticle-induced keratinocyte toxicity and exacerbation of allergic contact dermatitis. *HSOA J Toxicol* **4**, doi:10.24966/tcr-3735/100019 (2020).

2 Palmer, B. C., Jatana, S., Phelan-Dickinson, S. J. & DeLouise, L. A. Amorphous silicon dioxide nanoparticles modulate immune responses in a model of allergic contact dermatitis. *Sci Rep* **9**, 5085, doi:10.1038/s41598-019-41493-7 (2019).
